# Fabrication and Characterization of Inverted Silicon Pyramidal Arrays with Randomly Distributed Nanoholes

**DOI:** 10.3390/mi12080931

**Published:** 2021-08-05

**Authors:** Yue Zhao, Kaiping Zhang, Hailiang Li, Changqing Xie

**Affiliations:** 1 Key Laboratory of Microelectronic Devices & Integrated Technology, Institute of Microelectronics of Chinese Academy of Sciences, Beijing 100029, China; zhaoyue@ime.ac.cn (Y.Z.); zhangkaiping@ime.ac.cn (K.Z.); xiechangqing@ime.ac.cn (C.X.); 2 University of Chinese Academy of Sciences, Beijing 100049, China

**Keywords:** anti-reflectivity, broadband, metal-assisted chemical etching, anisotropic wet etching

## Abstract

We report the fabrication, electromagnetic simulation and measurement of inverted silicon pyramidal arrays with randomly distributed nanoholes that act as an anti-reflectivity coating. The fabrication route combines the advantages of anisotropic wet etching and metal-assisted chemical etching. The former is employed to form inverted silicon pyramid arrays, while the latter is used to generate randomly distributed nanoholes on the surface and sidewalls of the generated inverted silicon pyramidal arrays. We demonstrate, numerically and experimentally, that such a structure facilitates the multiple reflection and absorption of photons. The resulting nanostructure can achieve the lowest reflectance of 0.45% at 700 nm and the highest reflectance of 5.86% at 2402 nm. The average reflectance in the UV region (250–400 nm), visible region (400–760 nm) and NIR region (760–2600 nm) are 1.11, 0.63 and 3.76%, respectively. The reflectance at broadband wavelength (250–2600 nm) is 14.4 and 3.4 times lower than silicon wafer and silicon pyramids. In particular, such a structure exhibits high hydrophobicity with a contact angle up to 132.4°. Our method is compatible with well-established silicon planar processes and is promising for practical applications of anti-reflectivity coating.

## 1. Introduction

Anti-reflectivity coating plays a key role in the development of modern optoelectronic devices and has found various interesting applications. For example, anti-reflectivity coating is highly desirable to improve the quantum efficiency of back-illuminated detectors [1], to reduce the optical losses of solar cells [2], to facilitate the efficient capture of near infrared light in night vision cameras [3], and to create additional electron-hole pairs in avalanche photodiodes [4,5]. Generally, there are two categories to suppress reflection [6], i.e., enhancing transmission and enhancing absorption. To realize enhanced transmission, coatings that rely on the thin-film interference are adopted. The thickness of the layer should be a quarter or half of the wavelength of the light. Considering the minimum reflectivity under the wide-range light spectrum, the introduction of a multilayer structure can improve performance. However, the design and manufacture process are complicated. Additionally, most of these anti-reflective coating solutions are expensive because they require vacuum technology, which could be problematic in mass production. To realize the enhanced absorption, micro-nano structures are employed to change the direction of light to reduce the reflection, and materials with special optical properties such as Si_3_N_4_ [7], SiO_2_ [8], TiO_2_ [9,10] and GaAs [11] can interact with photons. The micro-nano structures that realize surface anti-reflection have various forms, most of the structures are derived from the moth-eye structure that was first discovered by Bernhard in nature [12,13]. Based on the moth-eye structure, a series of anti-reflective micro-nano structures have been derived, such as nano-pillars [14], nano-cones [15], nano-holes [16], nano-wires [17,18], etc. [19,20]. There are the following two main methods to fabricate: (i) dry etching, including reactive ion etching (RIE) and chemically assisted ion beam etching [17]; and (ii) wet etching, which uses chemical etchant solutions such as alkaline and acidic solutions [21,22].

Silicon is one of the most important semiconductor materials for energy conversion due to its photovoltaic effect [2,16,23]. However, incident light cannot be completely reflected [24]. Light-trapping films can improve the absorption of their composite devices by modifying its surface microstructure in the wavelength range of 300–1100 nm [17,25,26]. Compared to bare silicon or silicon with other structures, silicon with pyramidal structures can have reduced reflectance and a simple and stable fabrication process [21,27]. Anisotropic wet etching [28] takes advantage of the fact that different crystal orientations of Si have different etching rates in alkaline solutions, and, thus, allows for the preparation of large-area micro-nano pyramidal array structures on the surface of monocrystalline silicon. In conventional structures, pyramids are randomly distributed and overlap each other [20,29], which affect the efficiency of reflection. When the light sources impinge the wafer, the reflected light path is confirmed because all the reflecting surfaces are at the same angle [28]. In other words, the conventional pyramidal structure has a limited light trapping performance, and silicon with a regular inverted pyramidal textured film applied can achieve a reflectance of about 14% [30]. The principle of Metal-assisted chemical etching (MacEtch) is simple [31,32,33], Au nano particles (NPs) act as catalysts to induce local oxidation in acidic solutions, forming pores underneath. The corrosion rate of silicon under Au NPs is much higher than that of uncovered silicon in a mixed solution composed of hydrogen peroxide and hydrofluoric acid [34]. As the Au NPs sink, porous or columnar silicon nanostructures are formed. MacEtch can be used to generate deep structures, such as nanowires or pore structures, with a large aspect ratio [35]. Vertically aligned arrays of silicon nanostructures can provide nearly ideal photon absorption, thus reducing the size of semiconductor absorbers [16]. However, there is still the problem of a single direction of the reflected light path.

Here, an inverted pyramidal array with deep etched holes on a silicon wafer is realized by designing a fabrication process that combines the advantages of both etching methods. After conventional lithography on the silicon substrate, anisotropic wet etching in a KOH solution is performed to form the inverted pyramidal array. The random distribution of holes on the surface increases the structure density and aspect ratio, and this structure facilitates multiple reflection and absorption of light, thus improving the reflection efficiency. Since the intrinsic band gap of silicon is 1.12 eV, the general black silicon mainly concentrates on suppressing the reflection of light at wavelengths below 1100 nm [36], and the above structure ensures low reflectance in the visible region. Therefore, this structure can achieve a large surface area via a simple method with no toxic and harmful by-products and has good compatibility with the well-established silicon planar processes.

The reflectance is related to the permittivities at the surface; in 1904, Maxwell Garnett developed a simple but immensely successful homogenization theory. As any such theory, it aims to approximate a complex electromagnetic medium such as a colloidal solution of gold micro-particles in water with a homogeneous effective medium. The Maxwell Garnett mixing formula gives the permittivity of this effective medium in terms of the permittivities and volume fractions of the individual constituents of the complex medium. For anti-reflection structures such as nanostructures and pyramids, corresponding optical models can be established for auxiliary analysis through the effective medium analysis [37]. For the anti-reflection structure, the effective medium analysis regards the entire anti-reflection structure as a combination of multiple media. The Maxwell Garnett theory starts from the macroscopic Maxwell’s equations, which are assumed to be valid on a fine scale inside the composite.

## 2. Materials and Manufacturing Methods

**Figure 1:**
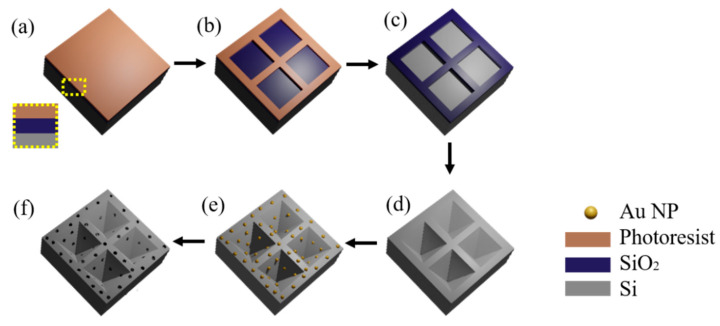
Schematic diagram of an inverted pyramidal array with deep etched holes using anisotropic wet etching and MacEtch process. (**a**) Oxidized silicon wafers substrate with photoresist NR1500; (**b**) Photo lithography; (**c**) Reactive ion etching (RIE) etching and remove the photoresist; (**d**) Anisotropic wet etching; (**e**) Deposition of Au layer and annealed to obtain random Au NPs; (**f**) MacEtch process.

The process flow for fabricating inverted pyramidal arrays with deep etched holes is shown in Figure 1. In our experiments, a 4-inch n-Type <100> wafer with 100-nanometer silicon oxide was used as substrate. The photoresist NR1500 was spin-coated onto the SiO_2_ with a thickness of 300 nm and then baked on a hot plate at 180 °C for 2 min (Figure 1a). The photoresist was exposed with a photolithograph (Suss MA6, Karl Suss, Germany) for pattern definition (Figure 1b) to form a square array. Reactive ion etching (RIE) was performed to transfer the resist pattern to the SiO_2_ layer, forming an Si square array exposed on the surface (Figure 1c).

An anisotropic wet etching process was used to generate an inverted pyramidal structure on (100) planar silicon (Figure 1d), which was etched at 85 °C in a solution containing DI water and KOH (30%), and the size of the resultant pyramid was determined by the etching time. For example, the etching time for a pyramid with side length of 6 μm in an alkaline etching solution is about 20 min. After etching, SiO_2_ was removed with a 10% HF solution.

A 4-nanometer Au thin film was deposited on the surface by a magnetron sputtering system (ACS-4000, ULVAC Company, Kanagawa, Japan). The working pressure was maintained at 4.5 × 10^−6^ Torr, and the temperature of the chamber was kept at 25 °C during the deposition process. After annealing at 300 °C for 3 min in air, Au NPs are obtained randomly on the sidewall of the inverted pyramid cavity and the surface of the silicon wafer with an average size of 6 nm (Figure 1e).

Then, MacEtch process was carried out to generate etching holes on the surface (Figure 1f). At room temperature, MacEtch takes place in 10 mL of hydrogen peroxide (H_2_O_2_, 30%), 10 mL of Hydrofluoric acid (HF, 49%) and 100 mL of DI water. Additionally, the depth of the corrosion hole is related to the etching time.

## 3. Results and Discussion

**Figure 2:**
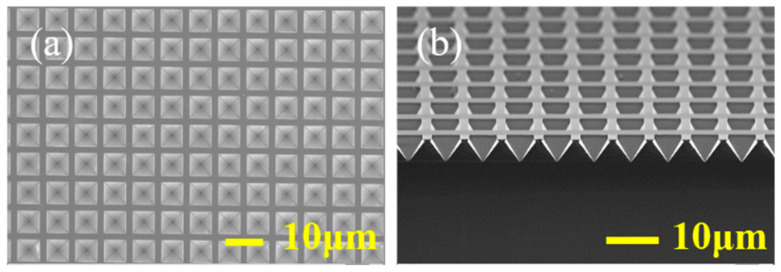
SEM images of the inverted pyramidal array images using anisotropic wet etching (**a**) top view, (**b**) cross section view.

**Figure 3:**
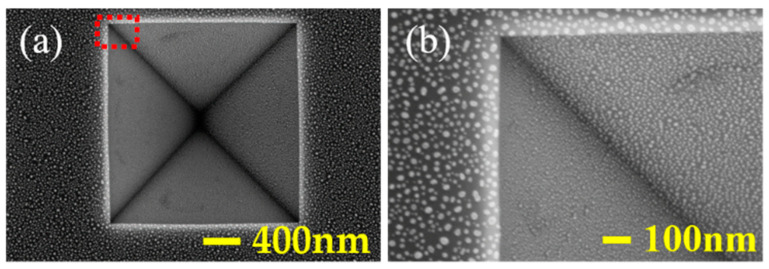
SEM images of randomly distributed Au NPs after annealing (**a**) top view, (**b**) zoomed in view of Au NPs at selected positions in (**a**).

The silicon structure of the inverted pyramidal array after anisotropic wet etching was observed using scanning electron microscopy (SEM) (ZEISS, SUPRA-55, Oberkochen, Germany). Figure 2a shows the SEM image of the top view of the silicon pyramidal array. The inverted pyramidal array has a period of 8 μm and a side length of 6 μm. The corresponding cross-section SEM image of the silicon pyramidal array is shown in Figure 2b. The angle between the sidewall and the bottom surface is 54.74° (the angle between the (111) plane and the (100) plane of monocrystalline Si) and the depth is 4.1 μm. In order to observe the whole pyramid and the Au NPs of such a small size at the same time, we take a pyramid with a side length of 2 μm as an example. Figure 3a shows that Au NPs are randomly distributed on the surface of silicon after annealing, with an average size of 6 nm. Figure 3b is zoomed in view of Au NPs at selected positions in Figure 3a.

**Figure 4:**
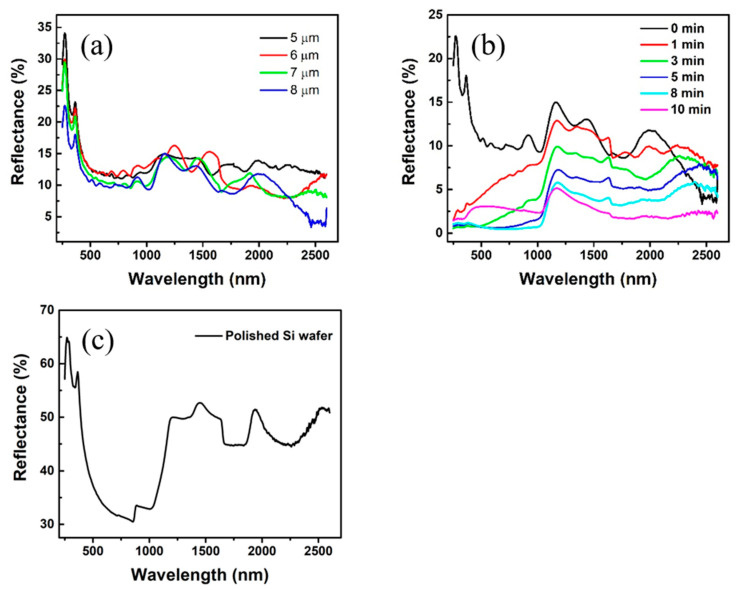
Reflectance spectra from 250 nm to 2600 nm for annealed inverted pyramidal arrays (**a**) with periods of 5, 6, 7 and 8 μm before MacEtch process; (**b**) with period of 8 μm treated by the MacEtch processes for 0, 1, 3, 5, 8, 10 min; (**c**) Specular reflection for polished Si wafer from 250 nm to 2600 nm.

We measured the reflectance of the as-prepared samples with a UV–vis–NIR Spectrophotometer (SHIMADZU, UV3600plus) equipped with an integrating sphere with 2.0-nanometer spectral bandwidth. The inner diameter of the integrating sphere is 60 mm, and the inner layer material is BaSO_4_. The light measuring range is 250–2600 nm. Before testing the samples, we used BaSO_4_ as a white board to perform the baseline calibration. After that, the sample is fixed at the corresponding position, and the diffuse reflection is performed. Indeed, there was a tiny amount of transmitted NIR light reflected and diffused back into the integrating sphere. It was not considered in this article. We measured the reflectance of annealed inverted pyramidal arrays with side lengths of 3, 4, 5 and 6 μm, respectively. The corresponding periods are 5, 6, 7 and 8 μm, respectively (Figure 4a). The reflectance decreases slightly as the size increases with a wavelength below 1100 nm. Since the angle of the etched surface is fixed, the depth will increase as the size of the inverted pyramid increases, the surface area will be larger, and the number of Au NPs will be more. Light will be trapped more efficiently in larger size pyramids. This morphology ensures the low reflectance, which could be attributed to gradient refractive index and multiple scattering effects. However, there is an increase in the NIR region near 1100 nm. This phenomenon is caused by the intrinsic bandgap of silicon (1.12 eV). There is no obvious regularity on the reflectance over 1100 nm of different sizes of the inverted pyramids. The average reflectance (250–2600 nm) of the pyramidal arrays with four different sizes are 13.6, 12.4, 11.4 and 10.5%. As we can see the average reflectance decreases slightly as the size increases over 1100 nm. We also measured the reflectance of polished Si wafer (Figure 4c) as a control, but samples with smooth surfaces such as mirrors and glass have almost no diffuse reflection. As the diffuse reflection is almost 0%, polished Si wafer is suitable for measurement in specular reflection mode. The average reflectance of polished Si wafer at broadband wavelength (250–2600 nm) is 44.6%.

**Figure 5:**
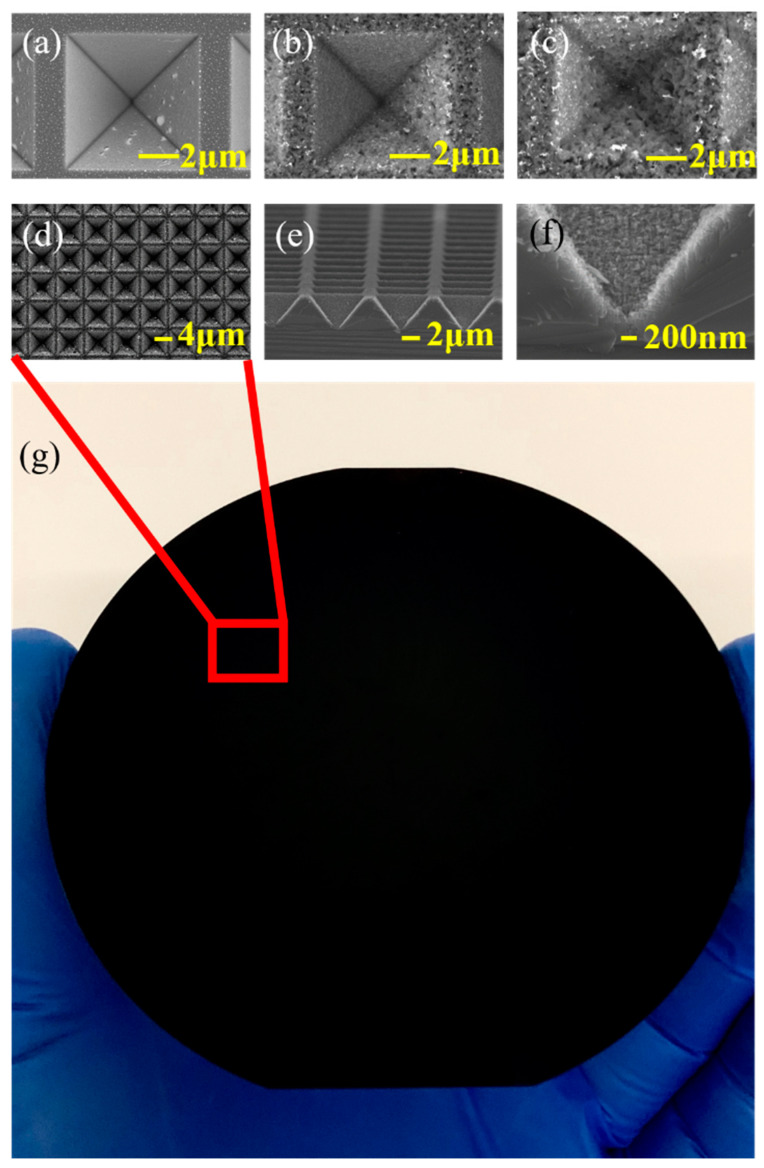
SEM images of inverted pyramids with period of 8 μm treated by the MacEtch processes for (**a**) 3 min; (**b**) 8 min; (**c**) 10 min; SEM micrographs of (**d**) top view; (**e**) side view; (**f**) enlarged view of inverted pyramids with period of 8 μm treated by the MacEtch processes for 8 min; (**g**) wafer-level fabrication of inverted silicon pyramidal arrays with randomly distributed nanoholes with a high wafer yield of Si anti-reflectivity coating.

We take the sample with an 8-micrometer period as an example: after the inverted pyramidal array with Au NPs were immersed in the etching solution, we observed the resultant morphology of inverted pyramids with different treating times of MacEtch (i.e., 3, 8 and 10 min, Figure 5a–c). As the etching time increases, both the etching depth and the surface roughness increase. It can be observed directly that the surface of the wafer gradually loses its luster and becomes darker due to the larger photon absorption. From 10 min onwards, the surface of the wafer becomes a grayish white. As the time increases, the etched holes are merged due to continuous expansion, the gap between the two adjacent inverted pyramids decreases. In this way, the reflection efficiency begins to decrease (Figure 4b). It can be inferred that the holes produced by MacEtch have made a great contribution to reducing reflectance. The anti-reflection performance in the NIR region (1100–2600 nm) is improved after MacEtch, with an average reflectance decreased from 10.5 to 4.40% at 8 min and 3.1% at 10 min. Taking a sample treated with 8 min MacEtch as an example, it can be seen from the top view, side view and enlarged view (Figure 5d–f) that the etched holes are almost perpendicular to the surface of the silicon substrate, and the average depth is 200 nm. The average reflectance at broadband wavelength (250–2600 nm) is 3.1%, which is 14.4 times lower than that of the polished Si wafer. As shown in Figure 5g, the method can be simply applied to a wafer-level fabrication of inverted silicon pyramidal arrays with randomly distributed nanoholes with a high wafer yield of Si anti-reflectivity coating. This demonstration indicates that our method is an effective way for the mass production of an Si anti-reflectivity coating with excellent anti-reflectivity performance.

In order to explain this phenomenon, we refer to the Maxwell Garnett model. Nanoscale surface structures, such as nanowire, nanorod and nano cone, provide enhanced absorption properties via anti-reflective and light scattering effects and offer a large junction area for charge separation and radial junction architecture. The further increase in the number of small structures on the side walls reduces the reflectance, demonstrating the efficient light trapping performance of the hierarchical structure. It is well known that the working principle of a superior light trapping structure is to efficiently complement the mismatch of the refractive index between the air and the bulk silicon, while for the hierarchical structure, since relatively small structures exist on the side walls, the mismatch between air and silicon obtain a better improvement and, thus, an improved gradual change of the refractive index profile occurs. Therefore, the hierarchical structure can provide a much lower reflectance than that of the other structures [37]. The Maxwell Garnett model regards the silicon material as the main material of the composite *ε_m_*, the air in the gap as the filling material *ε_i_* and the effective permittivity *ε_eff_* of the composite can be calculated using the following equation according to Maxwell Garnett mixing formula [38]:

(1) $$\frac{\varepsilon_{eff}-\varepsilon_{m}}{\varepsilon_{eff}+\varepsilon_{m}}=\delta_{i}\frac{\varepsilon_{i}-\varepsilon_{m}}{\varepsilon_{i}+2\varepsilon_{m}}$$

where *ε_eff_*, *ε_m_* and *ε_i_* are the effective permittivity of the composite, the permittivity of the primary material and the permittivity of the secondary material and *δ_i_* is the volume fraction of the secondary material in the composite.

According to the wave equation, Equation (2) shows that the reflectance is related to the permittivity of the media at the boundary as follows:

(2) $$R={\left|\frac{\sqrt{\varepsilon_{eff}}-1}{\sqrt{\varepsilon_{eff}}+1}\right|}^{2}$$

**Figure 6:**
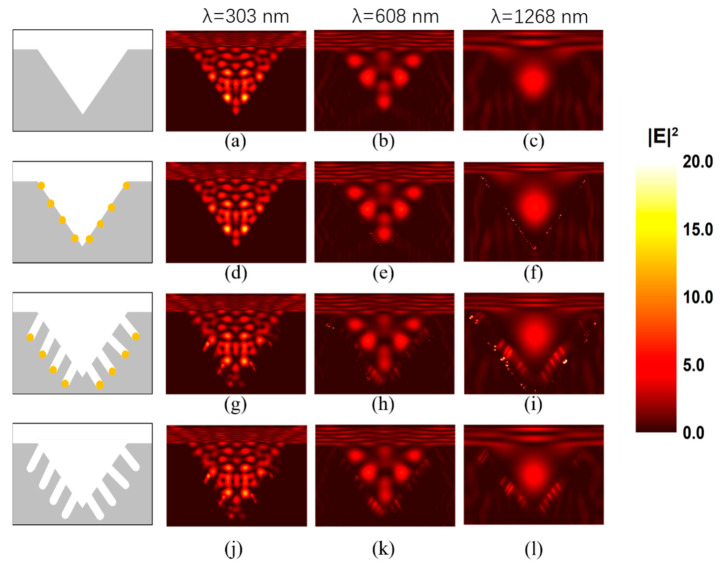
FDTD simulation of λ = 303, 608, 1268 nm with corresponding morphologies. (**a**–**c**) Simulation of an inverted pyramid without AuNPs at λ = 303, 608, 1268 nm; (**d**–**f**) Simulation of an inverted pyramid with AuNPs at λ = 303, 608, 1268 nm; (**g**–**i**) Simulation of an inverted pyramid with nanoholes and AuNPs at λ = 303, 608, 1268 nm; (**j**–**l**) Simulation of an inverted pyramid with nanoholes but without AuNPs at λ = 303, 608, 1268 nm.

According to Maxwell’s equations, light intensity is proportional to the square of the electric field intensity; therefore, the electric field intensity can explain the distribution of the light. We used FDTD to simulate the electromagnetic enhancement phenomenon and observe the internal electric field. To simulate the electric field intensity distribution, we built an inverted pyramid to simplify the model. We simulated the electric field distribution at 250–2600 nm in the following three situations: ① an inverted pyramid without AuNPs; ② an inverted pyramid with AuNPs; ③ an inverted pyramid with nanoholes and AuNPs; ④ an inverted pyramid with nanoholes but without AuNPs. The schematics are in the first column in Figure 6. The black frame in the schematic diagram is the simulation area, the gray part is Si, the white part is air, and the golden part is AuNP. The boundary conditions in the x and y directions were applied to periodic boundary conditions. The perfect matching layer was applied in the z direction. The incident light was set as a plane wave along the z direction. As shown in Figure 6b,d,f, the distribution plot of the cross-section view show that “hot spots” were formed in the inverted pyramid cavity at the wavelength λ = 303, 608 and 1268 nm, which were selected from UV, visible and NIR regions, respectively. The side length of the pyramid is 2 μm, the diameter of the AuNPs is 10 nm and the depth of the holes is 200 nm. The electromagnetic field in the concave structure is higher than the surrounding Si. It demonstrated that the distribution of light (electromagnetic waves) is greatly influenced by the designed structure.

**Figure 7:**
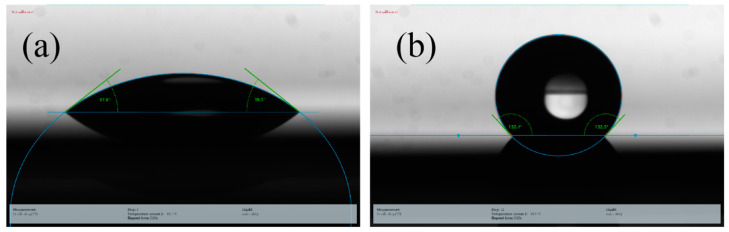
Photographs of the contact angle of a 5-microliter water drop (**a**) on bare silicone, (**b**) on an 8-micrometer inverted pyramidal array after being treated with the MacEtch process for 8 min.

At the same time, the reflected or scattered light is weakened again on the other sidewalls through the interaction with Au NPs on the surface. We add several Au NPs on the surface of the inverted pyramid in the model in Figure 6d–f. Compared with Figure 6a–c, a V shape electric field intensity enhancement can be observed, which were the same shape as the surface of inverted pyramids. Compared with Figure 6a–f, Figure 6h–g proved that it is indeed the holes produced by the AuNPs that have an obvious anti-reflection effect. From the comparison of Figure 6h–g, we find that the electric field of AuNPs are getting stronger and stronger as the wavelength increases. Therefore, the electric field was enhanced on the surface caused by Au NPs and randomly distributed nanoholes. In addition, we simulated a pyramid with nanoholes but without AuNPs (Figure 6j–l) to explore the influence of nanoholes and AuNPs on the electric field. It can be seen that both nanoholes and AuNPs have a certain influence on electric field intensity. However, in the actual structure, the volume of nanoholes or pores is larger than AuNPs. The simulation is a relatively simplified situation and serves as a reference. The nanoholes contribute more to the reflectance, while AuNPs also help to reduce the reflectance. Therefore, it can be inferred that this structure reduces the reflectance, which is the synergistic effect of the inverted pyramidal structure obtained using anisotropic wet etching, the porous structure formed through the MacEtch process and the Au NPs. In addition, this structure not only achieves a reduction in reflectivity, but also has self-cleaning properties. This self-cleaning capability maintains the anti-reflective properties when contaminated with dust particles in an outdoor environment. Figure 7b shows the contact angle (KRÜSS GmbH, DSA30E, Hamburg, Germany) of a 5-microliter water drop on an 8-micrometer inverted pyramidal array after being treated with the MacEtch process for 8 min, which exhibits hydrophobicity by increasing the contact angle from 39.3 to 132.4° compared to polished Si wafer (Figure 7a). There is a 2-nanometer unavoidable oxide layer and residual organic matter on polished Si wafer. Therefore, the inverted pyramids with deeply etched holes can take advantage of the self-cleaning behavior induced by the hydrophobic surface properties in addition to its anti-reflective properties.

## 4. Conclusions

In summary, an inverted pyramidal array with randomly distributed nanoholes was prepared on a silicon substrate using a combination of anisotropic wet etching and MacEtch, which has a strong anti-reflection capability. This morphology facilitates the multiple reflection and absorption of photons, thus improving the reflection efficiency. We investigated the effect of inverted pyramidal structure size and MacEtch time on reflectance. The structure exhibits excellent anti-reflective properties. When the anisotropic wet etching time is 20 min and the MacEtch time is 8 min, the average reflectance of this structure in the UV region (250–400 nm), visible region (400–760 nm) and NIR region (760–2600 nm) are 1.11, 0.63 and 3.76%, respectively. The resulting nanostructure can achieve the lowest reflectance of 0.45% at 700 nm and the highest reflectance of 5.86% at 2402 nm. The reflectance at broadband wavelength (250–2600 nm) is 14.4 and 3.4 times lower than silicon wafer and silicon pyramids. We used FDTD to simulate the electromagnetic enhancement phenomenon and observed the internal electric field. It demonstrated that the distribution of light depends on the designed structure. In particular, such a structure exhibits high hydrophobicity with a contact angle up to 132.4°. We expect that such an efficient fabrication method can find widespread applications in photo detectors sensitive layers for luminescence and other optoelectronic devices.

## Data Availability

All data generated or analysed during this study are included in this published article.
